# Single-cell transcriptomic analysis of honeybee brains identifies vitellogenin as caste differentiation-related factor

**DOI:** 10.1016/j.isci.2022.104643

**Published:** 2022-06-18

**Authors:** Wenxin Zhang, Liangliang Wang, Yinjiao Zhao, Yufei Wang, Chaoyang Chen, Yu Hu, Yuanxiang Zhu, Hao Sun, Ying Cheng, Qinmiao Sun, Jian Zhang, Dahua Chen

**Affiliations:** 1 Institute of Biomedical Research, Yunnan University, Kunming, Yunnan 650500, China; 2 School of Life Sciences, Yunnan University, Kunming, Yunnan 650500, China; 3 State Key Laboratory of Membrane Biology, Institute of Zoology, Chinese Academy of Sciences, Beijing 100101, China; 4 Institute of Stem Cells and Regeneration, Chinese Academy of Sciences, Beijing 100101, China

**Keywords:** Entomology, Genetics, Omics

## Abstract

The honeybee (*Apis mellifera*) is a well-known eusocial insect. In honeybee colonies, thousands of sterile workers, including nurse and forager bees, perform various tasks within or outside the hive, respectively. The queen is the only fertile female and is responsible for reproduction. The queen and workers share similar genomes but occupy different caste statuses. We established single-cell transcriptomic atlases of brains from queens and worker subcastes and identified five major cell groups: Kenyon, optic lobe, olfactory projection, glial, and hemocyte cells. By dividing Kenyon and glial cells into multiple subtypes based on credible markers, we observed that vitellogenin (*vg*) was highly expressed in specific glial-cell subtypes in brains of queens. Knockdown of *vg* at the early larval stage significantly suppressed the development into adult queens. We demonstrate *vg* expression as a "molecular signature" for the queen caste and suggest involvement of *vg* in regulating caste differentiation.

## Highlights

• scRNA-seq revealed distinct gene expression in the brains of queens and workers
• Vitellogenin (*vg*) may represent a "molecular signature" of the queen caste
• Knockdown of *vg* at early larval stage suppressed development into adult queens
• *Vg* may be involved in regulating caste differentiation

## Graphical abstract

**Figure:**
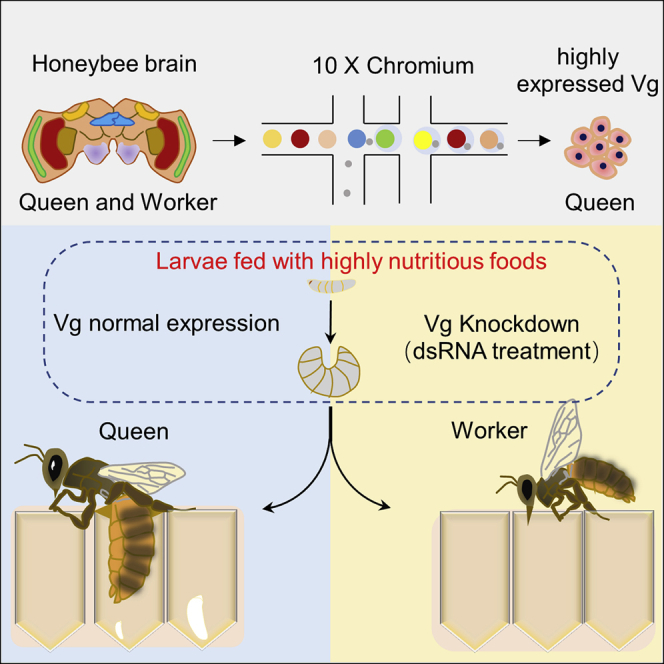

## Introduction

The honeybee is a well-known eusocial insect. In a colony of honeybees, the queen is primarily responsible for reproduction, whereas the workers are sterile and perform tasks required for the colony’s growth, maintenance, and defense (Johnson, 2010; Page et al., 2012). Workers usually perform tasks based on an age-related (behavioral-developmental) process (Beshers and Fewell, 2001; Calderone, 1998; Robinson and Huang, 1998). In the first two to three weeks following eclosion, an adult worker is responsible for the nursing tasks (e.g., caring for the brood and queen); the worker subsequently switches to other specialized work for a few days, including building and guarding within the hive. Eventually, the role of the worker changes to foraging for foods such as nectar, pollen, and water, for the rest of its life (Ament et al., 2010; Beshers et al., 2001). This behavioral switch is common but not rigid. Instead, honeybees are sensitive to environmental changes and duties are adjusted, reflecting typical phenotypic plasticity (Duncanm et al., 2020; Maleszka, 2018; Sommer, 2020).

Queens and workers develop from diploid fertilized eggs, and therefore share similar genomes. However, they exhibit tremendous differences regarding behaviors and physiologies. The process involved in formation of the two castes is termed caste differentiation, which largely depends on feeding conditions during the early larval stage (within the first 96 h) (Kamakura, 2011; Wang and Li-Byarlay, 2015; Weaver, 1966). However, the molecular basis of the establishment and regulation of caste differentiation remains largely unknown. Previous studies proposed that nutritional conditions during early development profoundly influence the individual’s epigenetic status (e.g., DNA methylation (Foret et al., 2012; Kucharski et al., 2008; Lyko et al., 2010), RNA m6A modification (Wang et al., 2021), and histone acetylation (Spannhoff et al., 2011)) and, thereby, determine the caste fate of the honeybee. In addition, nutrition-related signaling pathways, such as the insulin/insulin-like signaling (Nijhout and McKenna, 2018; Wang et al., 2013; Wheeler et al., 2006) and target of rapamycin nutrient-sensing pathways (Mutti et al., 2011; Patel et al., 2007; Wheeler et al., 2014), have been reported to participate in caste differentiation regulation.

Previous studies investigating neuronal cells in the bee brain suggested that there are remarkable differences in the transcriptomes (Behura and Whitfield, 2010; Liu et al., 2011, 2012; Tholken et al., 2019), proteomes (Hernandez et al., 2012), and methylomes (Foret et al., 2012; Herb et al., 2012; Lyko et al., 2010) between workers and queens. To better understand the difference between queens and workers, we established high-quality single-cell transcriptomic atlases of female honeybee brains. We identified a specific cell type in the brains of queens—ensheathing glial cells that highly express vitellogenin (*vg*). The expression of *vg* in a subset of glial cells serves as a "molecular signature" for the queen caste. Vitellogenin is a conserved yolk precursor protein exhibiting pleiotropic functions in honeybees. It has been reported that honeybee *vg* is associated with many central biological processes, including lifespan, reproduction, and immunity (Amdam et al., 2004; Brandt et al., 2005; Guidugli et al., 2005). To test whether *vg* contributes to caste differentiation, we performed RNAi (RNAi) assays and found that the knockdown of *vg* at the early larval stage significantly suppressed the development from larvae to the adult queen when reared under high-nutrition conditions. Therefore, in addition to its previously identified role in behavioral transitions between nurses and foragers (Ihle et al., 2010; Nelson et al., 2007), our findings suggest that *vg* is involved in regulating caste differentiation.

## Results

### Single-cell transcriptomic atlases of honeybee brains

**Figure 1:**
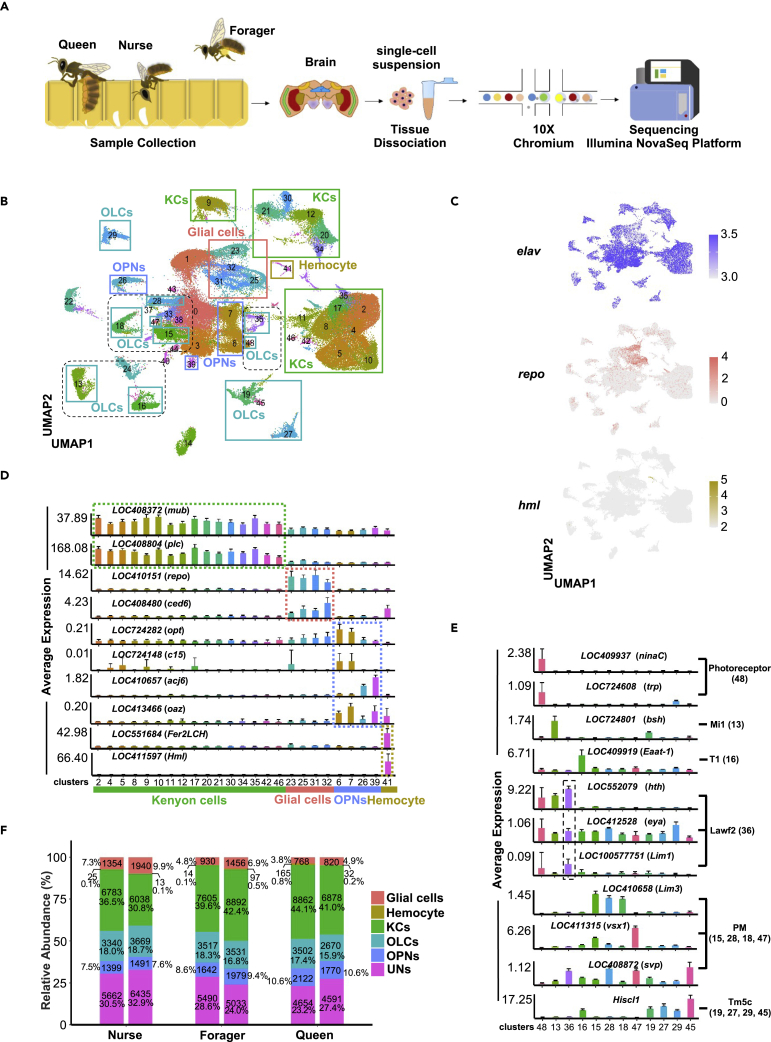
Single-cell transcriptomes of whole brains from queen, forager, and nurse bees (A) Scheme of the experimental design. Three female groups in honeybee colonies, including queens, nurses, and foragers, were collected based on behavior and morphological features. Brains were dissected and then dissociated into a single-cell suspension. The 10X single-cell RNA-seq libraries were processed for high-throughput sequencing. (B) Cell populations were identified. The UMAP projection of 115,169 filtered single cells from the brains of queens, nurses, and forgers shows the formation of five major clusters with label names. Each dot corresponds to a single cell, color coded according to cell type. KCs: Kenyon cells; OPNs: olfactory projection neurons; OLCs: optic lobe cells. (C) Canonical cell markers, *elav*, *repo*, and *hml*, were used to label neuron, glia, and hemocyte clusters, respectively, as represented in the UMAP plot. (D) Selected marker genes for Kenyon cells, glia, olfactory projection neurons, and hemocytes. The y axis shows the average expression in each cluster for the indicated gene. Bars represent the means of two biological replicates +SD. (E) Selected marker genes for the subtypes of optic lobe cells. The y axis shows the average expression in each cluster for the indicated gene. Bars represent the means of two biological replicates +SD. (F) Cell numbers and proportions of six major cell types among nurse, forager and queen brain samples. KCs: Kenyon cells; OLCs: optic lobe cells; OPNs: olfactory projection neurons; UNs: undefined neuron cells. Split bars showing the cellular composition of the replicates for each caste or subcaste. See also Figures S1 and S2, and Table S4. Predicted marker genes for all cell clusters, related to Figure 1, Table S5. Transcripts in Glial cells, related to Figure 1, Table S6. Transcripts in Kenyon cells, related to Figure 1, Table S7. Transcripts in Optic lobe cells, related to Figure 1., Table S8. Transcripts in Olfactory projection neurons, related to Figure 1, Table S9. Transcripts in Hemocytes, related to Figure 1, Table S10. Transcripts in Undefined neurons, related to Figure 1.

To identify caste-specific cell types among female honeybee brains, we employed 10X Genomics technology to conduct single-cell RNA-seq analysis. For this assay, we collected pools of four to five female honeybee brains from queens, foragers, and nurses, respectively. For each caste or subcaste, we sequenced two independent biological replicates, targeting over 10,000 cells per replicate. The 10X single-cell libraries were constructed, followed by high-throughput sequencing (Figure 1A). Following the recommended standard procedures, FASTQ files were analyzed using the Cell Ranger pipeline to generate feature-barcode matrices. A total of 115,169 cells were obtained after data filtration using the Seurat R package with three parameters: the nCount (number of unique molecular identifiers), nFeature (number of genes), and percent.mt (portion of mitochondrial genes) (Figure S1A). These cells included 40,186 (34.89%), 38,149 (33.12%), and 36,834 (31.98%) cells from the brains of foragers, nurses, and queens, respectively (Table 1). To validate the sensitivity and integrity of the single-cell dataset, we also performed bulk RNA-sequencing in parallel using corresponding samples. The comparison confirmed that the filtered single-cell data were highly correlated (r ≥ 0.7) to our bulk sequencing data (Figures S1B and S1C).

**Table 1:** Numbers of captured cells from different samples in single-cell RNA-seq

Cell type	Forager		Nurse		Queen	
	rep1	rep2	rep1	rep2	rep1	rep2
Glial cells	930	1456	1354	1940	768	820
Kenyon Cells	7605	8892	6783	6038	8862	6878
Olfactory Projection Neurons	1642	1979	1399	1491	2122	1770
Optic Lobe Cells	3517	3531	3340	3669	3502	2670
Hemocyte	14	97	25	13	165	32
Undefined neurons	5490	5033	5662	6435	4654	4591
Sum	19,198	20,988	18,563	19,586	20,073	16,761

We then integrated all the high-quality cell data into an unbatched and comparable dataset, which was then projected onto two dimensions via uniform manifold approximation and projection (UMAP) to visualize the cellular heterogeneity. As shown in Figures 1B and 1A total of 49 high-confidence cell clusters were identified. Of these, 37 clusters could be functionally annotated with marker genes predicted by FindAllMarkers (Table S4. Predicted marker genes for all cell clusters, related to Figure 1, Table S5. Transcripts in Glial cells, related to Figure 1, Table S6. Transcripts in Kenyon cells, related to Figure 1, Table S7. Transcripts in Optic lobe cells, related to Figure 1., Table S8. Transcripts in Olfactory projection neurons, related to Figure 1, Table S9. Transcripts in Hemocytes, related to Figure 1, Table S10. Transcripts in Undefined neurons, related to Figure 1) and orthologs of reported marker genes in *Drosophila*. Based on these findings, we divided the cells into five major categories: Kenyon cells (KCs), olfactory projection neurons (OPNs), glial cells, hemocytes, and optic lobe cells (OLCs) (Figures 1C–1E). Most cells were defined as neuron cells because they expressed the pan-neuron marker *elav* (Robinow and White, 1991), which is annotated as *LOC410689* in the honeybee. We recognized three main subgroups of neurons: KCs, OPNs, and optic lobe cells. Notably, KCs (clusters 2, 4, 5, 8–12, 17, 20, 21, 30, 34, 35, 42, and 46) were effectively distinguished by the enrichment of LOC408804 (*PLCe*) and *LOC408372* (*mub*) (Suenami et al., 2018), and OPNs (clusters 6, 7, 26, and 39) were identified by *LOC724282* (*otp*), *LOC724148* (*C15*), *LOC410657* (*acj6*), and *LOC413466* (*oaz*) (Davie et al., 2018); the highly abundant neurons of the optic lobe, collectively termed OLCs, were identified by a combination of markers. The captured OLCs were further divided into six subtypes: photoreceptors (cluster 48), Mi1 neurons (cluster 13), T1 neurons (cluster 16), PM neurons (clusters 15, 28, 18, and 47), Lawf2 neurons (cluster 36), and Tm5c neurons (clusters 19, 27, 29, and 45) (Davie et al., 2018). The corresponding markers are shown in Figure 1E.

In addition to the neurons, we found that cells expressing *LOC410151* (*rx2*), the homolog of *repo*, were recognized as glial cells (Edwards and Meinertzhagen, 2010; Shah et al., 2018). As a result, 7,268 (∼6.3%) glial cells were detected in our datasets, similar to the proportion found in *Drosophila* (∼6.4%) (Davie et al., 2018). Moreover, we also observed a few cells (∼0.3%, cluster 41) expressing the hemocyte markers *LOC411597* (*hml*) and *LOC551684* (*Fer2LCH*) that have also been found in the brains of fruit flies and ants (Davie et al., 2018; Sheng et al., 2020). Owing to the lack of marker genes enabling further subdivision, we termed the remaining 12 clusters "undefined neurons", because *elav* could be detected in these groups.

To compare differences in the cellular composition of brains derived from different castes, we counted the relative abundance of each cell category in the three female honeybee groups (Figures 1F, S2A and S2B). As shown in Figure 1F, the neuron compositions of queens and foragers exhibited similar patterns, and the ratios of KCs and OPNs were higher than those in nurses. Moreover, consistent results were obtained between the two biological replicates for each tested group.

### Neuron classification using neurotransmitters and neuropeptides

**Figure 2:**
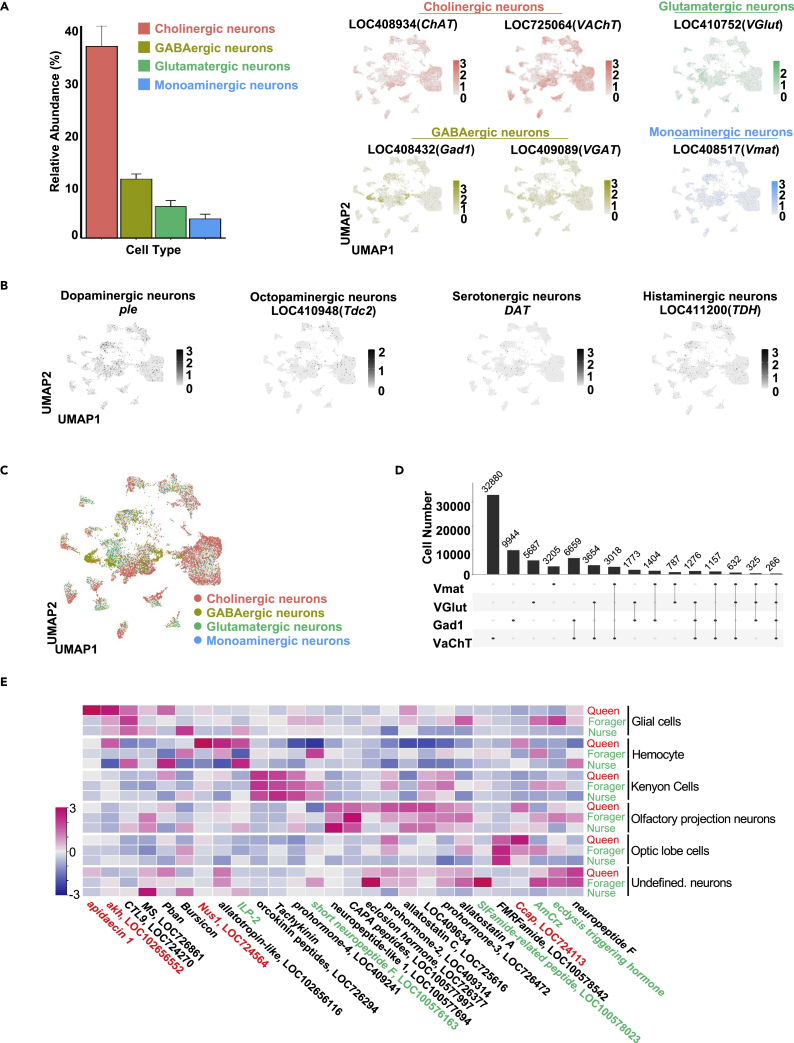
Expression of neurotransmitters and neuropeptides in the honeybee brain (A) Left panel: bar plot showing the relative abundance of cells in the honeybee brains assigned using different neurotransmitters. Bars represent the means of two biological replicates +SD Right panel: UMAP plots showing distribution of cells expressing biomarkers for the neurotransmitters including acetylcholine (*VAChT*, *ChAT*), glutamate (*VGlut*), GABA (*VGAT*, *Gad1*) and monoamine (*Vmat*). Expression shown in corresponding colors, intensity is proportional to the normalized expression levels. (B) UMAP plots showing sub-clustering analysis of *Vmat* positive cells. Four sub-clusters are identified representing dopaminergic (ple), octopaminergic (Tdc2), serotonergic neurons (DAT), and histaminergic (TDH). (C) UMAP plot color-code according to neurotransmitter expression. (D) UpSet plot (Conway et al., 2017) showing the co-expression of neurotransmitters. Numbers represent the number of cells. (E) Heatmap showing the expression of 27 indicated neuropeptides in different cell types of the three female groups. Expression was shown in magenta, intensity is proportional to the normalized expression levels. Queens (red) and workers (green) are distinguished. The caste-specific expressed neuropeptide genes are labeled with different colors. Red: queen-specific genes. Green: worker-specific genes. See also Figure S3.

During annotation, we observed that various genes associated with neurotransmitters and receptors were differentially expressed in the neuron subtypes. It has been demonstrated that neurotransmitters, including acetylcholine (ACh), glutamate, GABA, and monoamine molecules (e.g., octopamine and dopamine), are important in mediating distinct pathways involved in a variety of cognitive processes (Blenau and Baumann, 2001). On analyzing our datasets, we identified cholinergic (*VAChT*, *ChAT*), glutamatergic (*Gad1*, *VGAT*), and GABAergic (*VGAT*) neurons (Figure 2A), based on established markers that participate in the synthesis or vesicle transport of neurotransmitters (Allen et al., 2020; Brunet Avalos et al., 2019), accounting for 40, 8, and 15% of the total neurons, respectively. *Vmat* has been used to label monoaminergic neurons (Allen et al., 2020), and we found that *Vmat*-positive cells (∼10% of neurons) could be further divided into dopaminergic (*ple*), octopaminergic (*Tdc2*), serotonergic (*DAT*), and histaminergic (*TDH*) neurons (Figures 2A and 2B). Amongst the monoaminergic neurons, there were remarkably more dopaminergic neurons than the other three subtypes.

Previous studies reported the co-existence of more than one neurotransmitter in the same neuron in *Drosophila* adult and larval brain cell atlases (Brunet Avalos et al., 2019). To ascertain if this also applied to honeybee brains, we evaluated all possible combinations between the four markers *VAChT*, *Gad1*, *VGlut*, and *Vmat*. As shown in Figures 2C and 2D, nearly half of the neurons (51.9%) expressed one neurotransmitter, whereas the other neurons expressed two or more neurotransmitters. The statistical analysis revealed no significant differences in the neurotransmitter expression patterns among the three female groups (Figure S3A). Notably, the frequency of dual (17.3%) or multiple (3.7%) neurotransmitter-expressing neurons in the honeybee brains was significantly higher than that in fruit flies (10 and 1.8%, respectively) (Brunet Avalos et al., 2019), revealing the complexity of the neural communication network in honeybee brains.

Neuropeptides represent another type of essential messenger molecule for communication between neurons. Although numerous putative neuropeptides were previously observed in honeybees, the role of these peptides is poorly understood (Nassel and Homberg, 2006). We curated a panel of 27 reported honeybee neuropeptides and examined their expression at the single-cell level, and the heatmap in Figure 2E shows the cell-type-specific expression patterns of these neuropeptides in three female groups. Compared with results analyzed by bulk RNA-seq data (Figure S3B), single-cell RNA-seq data showed a detailed differential expression in each cell type. For example, tachykinin and FMRFamide were highly expressed in mushroom bodies and optic lobes (Figure S3C), which is consistent with previously reported findings (Schurmann and Erber, 1990; Takeuchi et al., 2004). Orcokinin, *CTL9*, and *ILP-2* were enriched in Kenyon cells, glia, and hemocytes, respectively. By comparing the datasets from the three female groups, we found that although most peptides exhibited a similar expression pattern, a few displayed caste-specific patterns. For instance, *Nus1* and *apidaecin 1* were highly expressed in the hemocytes and glial cells of queens, respectively, whereas *Crz* expression was specifically present in worker subcastes (Figure 2E).

### Reclustering of Kenyon cells

**Figure 3:**
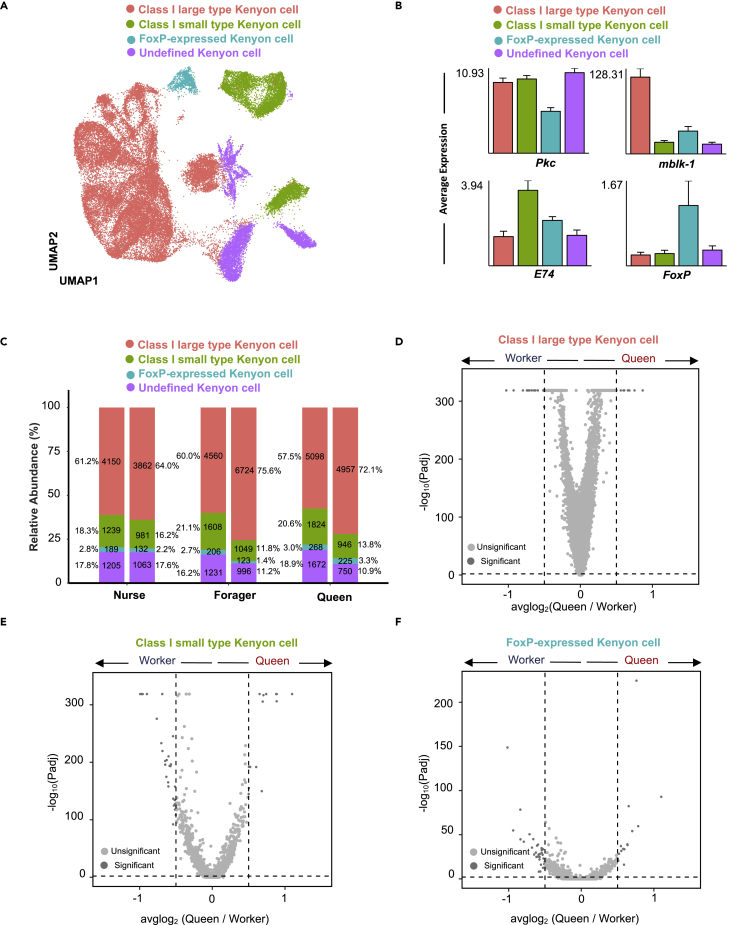
Reclustering analysis of honeybee Kenyon cells (A) UMAP plot of Kenyon cells, showing the composition of three identified subtypes. Each dot corresponds to a single cell, colored according to cell type. (B) Selected marker genes for Kenyon cell subtypes. The y axis shows the average expression in each cluster for the indicated gene. Bars represent the means of two biological replicates +SEM. (C) Average proportion of Kenyon cell subtypes among nurse, forager, and queen brain samples. The number of cells and the percentage of each cell type are shown. Split bars showing the cellular composition of the replicates in each caste or subcaste. (D–F) Volcano plot showing gene expression in Kenyon cell subtypes among queens and workers. The dashed line indicates log2FC threshold of +/−0.5. See also Figure S4 and Table S11.

Because KCs and glial cells are two major cell types in the honeybee brain, we then focused on elucidating their transcriptomic features. Kenyon cells of two major classes (class-I and class-II) located in the mushroom bodies are vital for sensory information processing, learning, and memory (Farris, 2005; Mobbs, 1982). Previous studies involving imaging analysis led to the class-I KCs being grouped into three subtypes: large (lKCs), middle (mKCs), and small (sKCs) (Suenami et al., 2018). By analyzing our single-cell seq datasets, we confirmed the existence of lKCs and sKCs with the specific markers *mblk-1* and *E74* (Suenami et al., 2016, 2018), respectively. Moreover, significant clustering of a KC population positively expressing *FoxP* (FoxP KCs) (Schatton and Scharff, 2017) was confirmed by our findings (Figures 3A, and 3B). In addition, class-II KCs failed to be defined because of a lack of information on suitable marker genes for bees. It is noteworthy that the three female castes shared a repertoire of closely related KC subtypes, although the proportion of all KCs in nurses was less than those in foragers and queens (Figure 3C).

We subsequently employed FindMarkers to analyze the differentially expressed genes (DEGs) in each KC subtype (Table S11) and found that the gene expression profiles of the lKCs, sKCs, and FoxP KCs between queen and worker castes were comparable on the volcano plot (Figures 3D to 3F). The Venn plots in Figure S4A summarize the number of DEGs in the two groups for each cell subtype. It appeared that there were considerably fewer DEGs in each KC subtype between nurses and foragers than between foragers and queens, which was in agreement with the bulk RNA-seq results (Figure S4B). However, the DEGs in these comparisons displayed no significant enrichment of gene ontology items.

### Reclustering of glial cells

**Figure 4:**
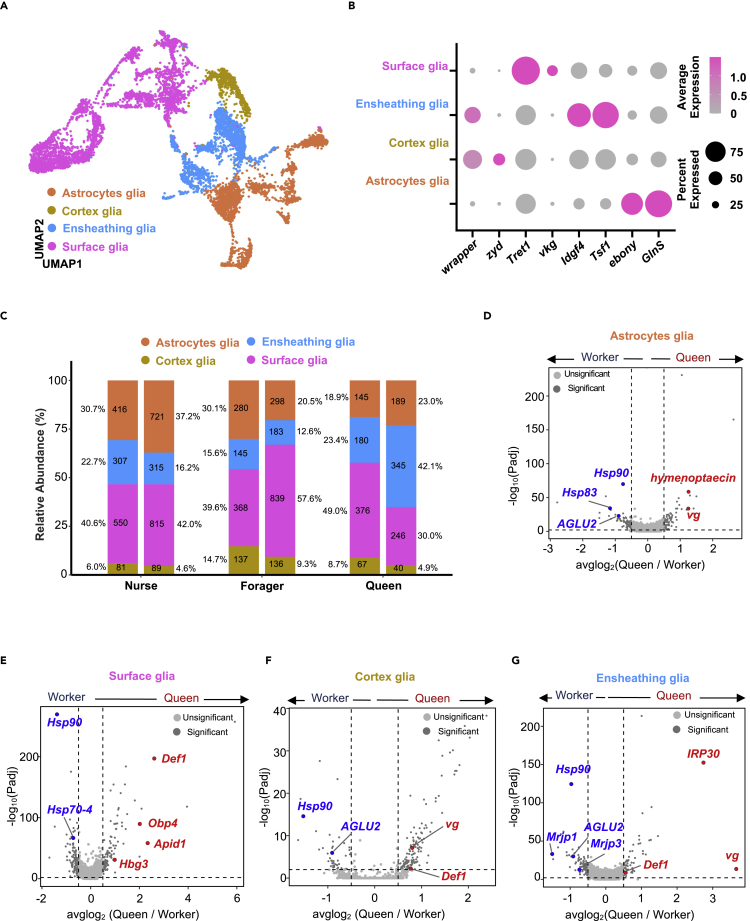
Reclustering analysis of honeybee glial cells (A) UMAP plot of glial cells, showing the composition of four identified subtypes. Each dot corresponds to a single cell, colored according to the cell type. (B) Dot plot showing the expression level and percentage of selected marker genes in different glia subtypes. Dot size corresponds to the percentage of cells expressing a particular gene, whereas color intensity represents gene expression levels. (C) Average proportion of glial cell subtypes among nurse, forager, and queen brain samples. The number of cells and the percentage of each cell type are shown. Split bars showing the cellular composition of the replicates in each caste or subcaste. (D–G) Volcano plots showing gene expression in each Kenyon cell subtype among queens and workers. The main DEGs with physiological functions are highlighted. The dashed line indicates log2FC threshold of +/−0.5. See also Figure S4 and Table S11.

Given that glial cells are essential for the functioning of the nervous system, we performed glial cell reclustering analysis by employing the marker genes used for classifying different subtypes of glia in *Drosophila* (Allen et al., 2020; Parker and Auld, 2006). As shown in Figures 4A and 4B, glia reclustering revealed four subtypes: surface glia (*Tret1*, *vkg*), astrocytes (*ebony*, *GlnS*), ensheathing glia (*Idgf4*, *Tsf1*), and cortex glia (*wrapper*, *zyd*). By calculating the proportion of each cell type, we identified different compositions among the three female groups (Figure 4C). In particular, the relative ratio of ensheathing glia was higher in queens' brains than in workers' brains. Ensheathing glial cells have been reported to function as phagocytes in the *Drosophila* brain (Doherty et al., 2009), and are also associated with the aging process (Sheng et al., 2020). We observed that nurse bees had relatively more ensheathing glia than foragers. Recent studies in *Harpegnathos* reported the expansion of ensheathing glia in the reproductive caste (Sheng et al., 2020). Hence, a high ratio of ensheathing glia could be a biomarker for the queen caste. In addition, DEGs in each glial cell subtype were depicted in volcano plots (Figures 4D–4G, Table S11), and the number of DEGs was summarized in Venn plots (Figure S4C).

### Ensheathing glia expressed *vg* in a queen-specific manner

**Figure 5:**
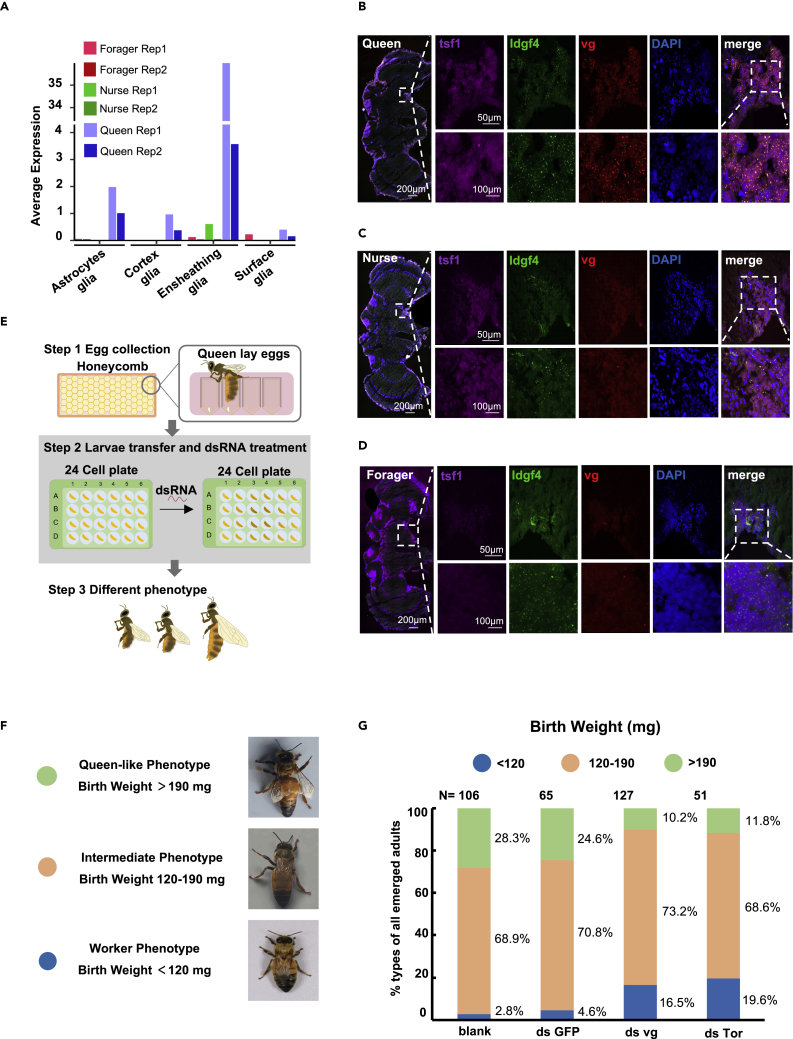
V*g* is expressed in ensheathing glia in a queen-specific manner and *vg* RNAi affects caste differentiation (A) Bar plot showing the average expression level of *vg* in four glia subtypes among replicates of nurses, foragers, and queens. (B–D) Simultaneous detection of *tsf1* mRNA (magenta), *idgf4* mRNA (green), *vg* mRNA (red), and (4′, 6-diamidino-2-phenylindole) DAPI-stained nuclei (blue), using confocal microscopy. The co-expression of *tsf1* and *idgf4* was used to mark ensheathing glia. Brain sections from the queen (B), nurse (C), and forager (D), respectively. Left: whole brain; Right: successively amplified regions with positive probe signals. Scale bars were indicated on the corresponding images. (E) Scheme of RNAi treatment assay based on the in-dish queen rearing system. First instar larvae were transferred into a 24-well plate and fed high nutritional foods. The double-strand RNAs were added or not added to the food in the treatment or control group, respectively. The pupae were transferred to another 24-well plate and kept still for eclosion. (F) The adults reared in the in-dish-rearing system are divided into three typical phenotypes according to birth weight. (G) Statistics for three phenotypes of four experimental groups (blank, ds GFP, ds vg and ds Tor). The phenotypes are colored according to different birth weight standards. The number of individuals and percentage of each phenotype are shown. See also Figure S5 and Table S12.

Having observed the high number of ensheathing glia in the brains of queens, we sought to analyze the differential gene expression between the ensheathing glia of queens and workers. As shown in Figures 4G and S4C, 111 genes were more highly expressed in the ensheathing cells of queens compared to those of workers (nurse and forager bees). In particular, ensheathing cells in queens displayed a log2-fold-change exceeding three in *vg* expression compared to the average expression levels of *vg* in worker ensheathing cells (Figure 5A). To confirm this observation, we performed fluorescence *in situ* hybridization (FISH) assays, using multiple probes including *vg*, *idgf4,* and *tsf1*. In this assay, *idgf4* and *tsf1* were used to specifically mark the ensheathing glial cells in adult brains. As shown in Figures 5B–5D, *vg* transcripts were highly expressed in ensheathing glial cells in queens' brains compared to that in the ensheathing glial cells in workers' brains. Although the expression of *vg* varied in honeybees at different ages, we confirmed that *vg* was significantly more highly expressed in the queen caste rather than in an age-dependent manner by using qPCR analysis (Figure S5A). As such, our results suggest that *vg-*positive ensheathing cells represent a specific subtype of glia in the queen brain and are likely to be a biomarker for the queen caste. Moreover, based on our findings, we then investigated whether *vg* contributes to caste differentiation.

Indeed, our bulk RNA-seq analysis also showed that *vg* was one of the most differentially expressed genes in the brains of queens and workers (Table S12). These results indicate that high expression of *vg* may play more important roles in queens compared to that in workers. Therefore, examining whether *vg* is involved in caste differentiation is an interesting issue that warrants exploration. Previous studies suggested that knocking down *vg* expression in adult workers affects the behavioral transition between nurses and foragers (Nelson et al., 2007). Given that caste differentiation is determined during the early larval stage, we knocked down *vg* throughout the larval stages by conducting RNAi on larvae kept in an in-dish rearing system (Figure 5E). In this assay, we transferred the first instar larvae to a 24-well plate and fed them with adequate high-nutrition food compounds for development. For RNAi treatment, we packaged double-strand (ds)RNAs within a previously described synthetic nanomaterial (Jian et al., 2009; Lynn and Langer, 2000), which was added to the bee food (Figure S5B). The bees were fed with dsRNA-treated food until the pupal stage. Pupae were then transferred to another 24-well plate (Figure S5C), and the emerged adults were used for phenotypic analysis.

There are numerous differences in the body structure of queens and workers (Ament et al., 2010). Queen bees can easily be recognized by specific morphological features, particularly birth weight and body size. In our in-dish-rearing system, three groups of adults with different body phenotypes developed (Figure 5F): (1) over 22% of adults appeared similar to natural queens (birth weight >190 mg), and we termed this group the "queen-like" bees; (2) ∼75% of adults were lighter than queens but heavier than workers (birth weight 120–190 mg), and we termed them "intermediate" bees; and (3) ∼3% of adults had body weights similar to those of workers (birth weight <120 mg), and we called this the "worker" phenotype. The worker phenotype was also confirmed at gene expression level using DEGs from bulk RNA-seq (Figure S5D to S). As shown in Figure 5G, *GFP* dsRNA treatment did not affect the ratios of the aforementioned types of adults in our cultured system. However, *vg* knockdown significantly reduced the percentage of queen-like bees and increased the ratio of workers in tested populations. *Tor* is a known factor involved in caste differentiation (Mutti et al., 2011). Interestingly, we found that *Tor* knockdown displayed similar results as *vg* knockdown (Figure 5G). Moreover, to test the role of *vg* at the early larval stage, we performed another RNAi assay in which *vg* was knocked down at the late larval stage (after 96 h) when the queen fate had already been established (Weaver, 1966). We did not observe any effects in this assay (Figure S5T), indicating that *vg* contributes to caste differentiation during the early larval stage. Therefore, our results reveal that *vg* knockdown at the early larval stage can affect the development trajectory of queens, highlighting the importance of *vg* expression in bee development.

## Discussion

Caste differentiation is a hallmark of a social insect community. The honeybee is an excellent model organism for studying the mechanisms underlying determination and regulation of the caste fate of queens. Previous studies proposed that nutritional conditions in coordination with epigenetic regulation and nutrition-related signaling pathways control caste differentiation (Kucharski et al., 2008; Mutti et al., 2011; Spannhoff et al., 2011; Wang et al., 2021). Given that the behaviors of animals in a colony are relatively inflexible, and the relatively complex behaviors are controlled by the brain (Leadbeater and Chittka, 2007), we speculated that caste determination might be accompanied by differentiation of specific cell types. To identify the difference between queen and worker castes, we performed transcriptomic analysis of different caste brains at the single-cell level, and we identified a specific subtype of ensheathing cell with high levels of *vg* expression in queen brains. In particular, *vg* was more highly expressed compared to the average *vg* expression level in the ensheathing cells of workers. Importantly, we found that the knockdown of *vg* at the early larval stage, but not the late stage, significantly suppressed the developmental trajectory to queens. Therefore, our study not only identifies the high expression of *vg* in a subset of glial cells as a "molecular signature," but it also suggests that *vg* is involved in caste differentiation.

By analyzing transcriptomic features in this study, we obtained an important finding that glial cell populations vary significantly between queen and worker castes. Specifically, we found that the ratio of ensheathing glia was most variable among the castes and subcastes. The ensheathing glia population has been reported to be associated with caste transition, injury, and aging in ants (Sheng et al., 2020). In addition, the function of the ensheathing glia has been extensively studied in other model organisms, including *Drosophila* and mammals (Freeman, 2015; Ramon-Cueto and Avila, 1998). Our findings further emphasize the importance of this type of cell in regulating development and behavior.

*Vitellogenin* is a well-studied factor that is involved in a variety of biological processes, including reproduction, immunity, aging, and behavioral transition (Amdam et al., 2003; Havukainen et al., 2011; Ihle et al., 2015; Nelson et al., 2007; Seehuus et al., 2007). In particular, *vg* is synthesized by the fat body and then released to the hemolymph. Its expression in the hemolymph of queens has been reported to be much higher than that in workers (Barchuk et al., 2002; Guidugli et al., 2005). In this study, we identified a specific subtype of ensheathing glial cells that expresses *vg* at extremely high levels, suggesting that *vg* expression might be associated with the differentiation of this cell subtype. After knocking down *vg* with RNAi at an early larval stage, we found that the fate of queens was inhibited. In conclusion, our work identifies a subset of glial cells with *vg* high expression as a "biomarker" for the queen caste. It also suggests that *vg* plays a role in regulating caste differentiation in honeybees.

### Limitations of the study

There are some limitations to this study. First, from a technical standpoint, the single-cell atlas cannot completely cover all the cell types, and rare cell populations might be omitted. Second, available genomic information of honeybees is insufficient and has not been fully elucidated. As a result, ∼30% of captured cells in our atlases are functionally undefined. Third, the variation between replicates increased during reclustering analysis, particularly for glia subtypes, which was also an inherent issue of the approach of single-cell RNA-seq. It is easy to produce the variation when we capture those rare cell types. Nevertheless, the findings from our “FISH” assays provided experimental evidence to support our conclusions.

## STAR★Methods

### Key resources table

.

**Table:** 

REAGENT or RESOURCE	SOURCE	IDENTIFIER
**Biological samples**		

Brains from queens for single-cell RNA-seq, 2 replicates	This paper	N/A
Brains from nurses for single-cell RNA-seq, 2 replicates	This paper	N/A
Brains from foragers for single-cell RNA-seq, 2 replicates	This paper	N/A
Brains from queens for bulk RNA-seq, 2 replicates	This paper	N/A
Brains from nurses for bulk RNA-seq, 2 replicates	This paper	N/A
Brains from foragers for bulk RNA-seq, 2 replicates	This paper	N/A

**Chemicals, peptides, and recombinant proteins**		

D (-) Fructose	Sigma-Aldrich	Cat# F3510
D (+) Glucose	Sigma-Aldrich	Cat# G7021
Bacto yeast extract	Thermo Fisher	Cat# 288620
fresh royal jelly	This paper	N/A
Papain, Lyophilized	Worthington	Cat# LS003119
Sf-900™ III SFM	Thermo Fisher	Cat# 12658019
DPBS	Thermo Fisher	Cat# 14190144
Bovine Serum Albumin	Sigma	Cat# A1933
10% Tween 20 solution	Bio-Rad	Cat# 1610781
Elution buffer	QIAGEN	Cat# 19086
TE buffer	Thermo Fisher	Cat# 12090015
Nuclease-Free water	Thermo Fisher	Cat# AM9937
Sodium acetate	Sigma	Cat# S5636
1,4-butanediol diacrylate	Alfa Aesar	Cat# 32780
Polyoxymethylene	Aladdin	Cat#C104190
Formamide	Macklin	Cat#F6287
N, N-Dimethylethylenediamine	TCI	Cat# D0719
Tetrahydrofuran	Energy Chemical	Cat# W310075
DMSO	Sigma	Cat# 472301
Trizol	Thermo Fisher	Cat# 15596018
AceQ Universal SYBR mixture	Vazyme	Cat# Q511
DAPI	Thermo	Cat#62248
Tween®20	Sigma	Cat#P1379-1L
Antifade Mounting Medium	VECTASHIELD	Cat#H-1000
Stellaris® RNA FISH Wash buffer A	Biosearch	Cat#SMF-WA1-60
Embedding medium for frozen	Sakura	Cat#4583
Hiscript III RT SuperMix for qPCR(+gDNA wiper)	Vazyme	Cat#R323-01

**Critical commercial assays**		

Chromium Next GEM Single Cell 3' Kit v3.1	10X Genomics	Cat# 1000121
Chromium Chip G Single Cell Kit	10X Genomics	Cat# 1000120
Single Index Kit Set A	10X Genomics	Cat# 1000213
AHTS® Universal V8 RNA-seq Library Prep Kit for Illumina	Vazyme	Cat# NR605-02
Qubit dsDNA HS Assay Kit	Thermo Fisher	Cat# Q32854
NEBNext® Poly(A) mRNA Magnetic Isolation Module	New England BioLabs	Cat# E7490
SPRIselect beads	Beckman Coulter	Cat# B23318
TransScript® First-Strand cDNA Synthesis SuperMix	TransGen	Cat# AT301-03
T7 RiboMAX™ Express Large Scale RNA Production System	Promega	Cat# P1320

**Deposited data**		

Raw data	This paper	GEO: GSE184507
Code	This paper	https://github.com/erroxml/Honeybee-scRNA-seq
Raw micrograph data	This paper; Mendeley Data	https://doi.org/10.17632/xrd623n43s.1

**Experimental models: Organisms/strains**		

Honey bees (*Apis mellifera*)	Institute of Biomedical Research, Yunnan University (China)	N/A

**Oligonucleotides**		

Primers for dsRNA DNA templates synthesis, see Table S1	This paper	N/A
Primers for qRT-PCR, see Table S2	This paper	N/A

**Recombinant DNA**		

pAc5.1-Flag-EGFP	This paper	N/A

**Software and algorithms**		

Cell Ranger (v3.1.0)	10X Genomics	https://support.10xgenomics.com
Seurat (v3.1.5)	(Butler et al., 2018)	https://satijalab.org/seurat
FastQC (v0.11.9)	Babraham Bioinformatics	https://www.bioinformatics.babraham.ac.uk/projects/fastqc/
Trimmomatic (v0.39)	(Bolger et al., 2014)	http://www.usadellab.org/cms/?page=trimmomatic
STAR (v2.7.9a)	(Dobin et al., 2013)	https://github.com/alexdobin/STAR
RSEM (v1.3.1)	(Li and Dewey, 2011)	http://deweylab.github.io/RSEM/
DEseq2 (v1.32.0)	(Love et al., 2014)	http://www.bioconductor.org/packages/release/bioc/html/DESeq2.html
DAVID (v6.8)	(Huang da et al., 2009a, b)	https://david.ncifcrf.gov/
TBtools (v1.082)	(Chen et al., 2020)	https://github.com/CJ-Chen/TBtools
GraphPad Prism (v8.2.1)	GraphPad Software, La Jolla, CA	N/A
ZEN lite blue (v3.1)	Zeiss	https://www.zeiss.com.cn/microscopy/products/microscope-software/zen.html
Imaris (v9.0.1)	Oxford Instruments Group	https://imaris.oxinst.com/support/imaris-release-notes/9-0-0#9-0-1

### Resource availability

#### Lead contact

Further information and requests for resources and reagents should be directed to and will be fulfilled by the lead contact, Dahua Chen (chendh@ynu.edu.cn or chendh@ioz.ac.cn).

#### Materials availability

This study did not generate new unique reagents.

### Experimental model and subject details

#### Honeybees and brain sample collection

All the *Apis mellifera* colonies used in this study were kept in apiary supported by the institute of biomedical research, Yunnan University. We chose morphologically distinguishable nurses and foragers from the same colony. The workers those entered cells and nursed larvae were recognized as nurses, while the foragers could be easily identified by the yellow pollen loaded on their hind legs. The mated queens were obtained by artificial rearing and inseminating methods.

### Method details

#### Preparation for single-cell suspension

Before dissection, all bees were anesthetized by carbon dioxide. The whole brains of alive bees were dissected in ice-cold SF9-III medium for each biological replicate and immediately transferred to a pre-cooling tube containing SF9-III medium. Four to five brains were pooled. The dissociated brains were incubated with papain solution (Worthington, 1 μg/μL) at 35°C for 30 min, and briefly centrifuged (50 g, 10 s) to collect cell suspension and remove undigested tissue fragments. The cell suspension was centrifuged again (300 g, 5 min), and the pellet was washed twice with DPBS. Subsequently, the cell pellet was resuspended into 3 mL PBSB (DPBS+0.04% BSA) and filtered through a 20 μm pluriStrainer. An AO/PI Dual-fluoresces counting assay performed on the Countstar FL system detected cell counting and viability. The cell suspension (cell viability >95%) was adjusted to a final concentration of 700∼800 cells/μL.

#### 10x Genomics and sequencing

The single-cell 3′ gene expression libraries were generated following the manufacturer’s instructions (Chromium Next GEM Single Cell 3′ Reagent Kits v3.1 User Guide). Each sample was processed on an independent Chromium Next GEM Chip G with a target capture of 10,000 cells. The quality of libraries was analyzed by Agilent 2100 Bioanalyzer. The libraries were sequenced by Illumina Novaseq 6000 platform.

#### Bulk RNA-seq

Five adult brains were pooled and homogenized in the Trizol reagent to extract total RNA. mRNA was isolated using the Oligo d(T)25 magnetic beads (NEB). The purified mRNA samples went through the steps of fragmentation, random primers annealing, double-stranded cDNA synthesis, adaptor ligation, ligation product purification, and library amplification according to the manufacturer’s instructions (VAHTS® Universal V8 RNA-seq Library Prep Kit for Illumina, Vazyme). Finally, a sequencing library suitable for the Illumina® platform was obtained. The quality of libraries was analyzed by Agilent 2100 Bioanalyzer. The libraries were then sequenced by Illumina Novaseq 6000 platform.

#### In-dish rearing of honey bee queens

Healthy mated queens were caged in a clean frame to lay eggs for 3-6 h (depending on the number of eggs). Then released the queen and waited for eggs to hatch. Before larvae transfer, prepared larval diet: 55% fresh royal jelly, 5% glucose, 10% fructose, 1% yeast extraction, and 30% distilled water; mix thoroughly. The 1st instar larvae were transferred from the comb to sterile 24-well plates. Each larva was fed with a 250 μL pre-heated (31°C) larval diet once every two days. The plates filled with larvae were placed horizontally in the constant temperature and humidity incubator (at 35°C, 95% R.H.). The mature larvae were moved to new sterile 24-well plates for pupation. The plates filled with pupae were placed horizontally in another incubator (at 35°C, 75% R.H.). When the color of pupae turned dark utterly, adult honeybees would begin to emerge.

#### Fluorescent *in situ* hybridization

Day1: Bee brains were dissected to remove Compound eye and ocellus, embedded in O.C.T and frozen in −80°C ready for section. 30μm is an appropriate thickness for the bee brain section to maintain tissue shape. The sections were washed with PBST for 2 min twice and then fixed with 4% paraformaldehyde for 30 min. Washed for 5 min twice in PBST, and sections were dehydrated with gradient ascending methanol (25%, 50%, 75%, 100%) (5 min for each concentration on ice) when sections were treated in 100% methanol，-20°C overnight. Day2: Dehydrated sections were treated with gradient descending methanol (100%, 75%, 50%, 25%) to rehydrated, PBST washed for 5 min twice on ice. And proteinase K (10 μg/mL) for 3 min, washed with PBST 5 min twice, then fixed again with 4% paraformaldehyde for 1 h. Washed with PBST for 5 min thrice. And they were treated with wash buffer (wash buffer A: PBST = 1:1) for 10 min, wash buffer A (6 mL Stellaris® RNA FISH Wash buffer A, 21 mL DEPC H2O, 3mL Formamide) for 5 min twice, HYB-(10 mL 20x saline sodium citrate, 20 mL formamide, 10 mL DEPC H2O, 40 μL Tween®20) for 5 min at room temperature then 1h at 37°C. Add probes (1: 600) diluted with HYB-(Probes for FISH were shown in Table S3). Placed sections at 37°C in an incubator for 18 h. Day3: Probes were removed thoroughly, HYB- 30 min at 37°C, PBST 2 min twice, DAPI (0.5 μg/mL) 30 min at room temperature, PBST 10 min thrice. The sections were mounted with Antifade Mounting Medium. Image reconstruction was processed using confocal microscopy.

#### Confocal imaging

The whole-brain imaging was performed using Zeiss LSM 980 confocal microscopes with Plan-Apochromat 40×/1.3 oil objective. Z-stack and tile scan features were used to image the large, wavy surfaces of the brain slices. The resulting tiles were then stitched into a single large image (ZEN 3.1 Blue software, Zeiss), which enabled visualization of the whole brain at high resolution. Imaris software (Imaris 9.0.1, Bitplane) was used to visualize images in 3D.

#### dsRNA synthesis and RNAi treatment

dsRNAs were synthesized in an *in vitro* transcription reaction using T7 RiboMAX™ Express Large-Scale RNA Production System (Promega). The DNA templates (∼500bp) of dsRNAs were amplified from the queens' ovaries cDNA. The T7 promoter sequence was added to the 5′end of all the primers for DNA template amplification. The primers used in this study were listed in Table S1.

To promote the efficiency and specificity of gene silencing, three dsRNAs targeted to different gene regions were mixed in equal proportions and diluted to a final concentration of 1 μg/μL of total dsRNA. For one treatment on one 24-well plate filled with larvae, 2.4 μg dsRNA mixture and 8μL transfection polymer material (Poly[oxy-1,4-butanediyloxy(1-oxo-1,3-propanediyl)[[2-(dimethylamino)ethyl]imino](3-oxo-1,3-propanediyl)]) were diluted in 80 μL 25 mM NaOAc buffer (pH5.0), vortexed vigorously for 30 s and then incubated at room temperature for 20 min. Dropwise added 3 μL mixture to the region near the larva’s head. The tips should avoid touching larva. This treatment was begun on the day after 1st instar larvae transfer and repeated once a day until the larval stage ended.

#### Synthesis of transfection polymer

To a solution of 1,4-butanediol diacrylate (10 mmol, 1982.2 mg, 1.982 mL) in THF (20 mL), diamine (10 mmol, 881.5 mg, 1.092mL) was added. The reaction mixture was stirred at 50°C for 48 h. After that, the reaction was cooled to room temperature and concentrated under reduced pressure. The polymer was prepared with DMSO into a solution of 500 mg/mL.

#### Quantitative real-time PCR

Total RNA was isolated with Trizol Reagent (Invitrogen). cDNA was synthesized using TransScript® First-Strand cDNA Synthesis SuperMix (TransGen). Quantitative RT-PCR was performed using AceQ Universal SYBR mixture (Vazyme) in duplicate on LightCycler 480 Ⅱ (Roche). Template concentrations were normalized to endogenous reference RpL32. Primers for quantitative RT-PCR were designed by PerlPrimer and shown in Table S2.

#### 10x data preprocessing

The 10x honeybee brain samples were each processed by the Cell Ranger (version 3.1.0) count pipelines provided by 10X Genomics to generate single feature-barcode matrices. The reference genome was built based on *A. mellifera* (assembly Amel_HAv3.1) from NCBI.

#### Data processing by seurat

The feature-barcode matrices of each sample (two biological replicates were performed for each queen, forager, and nurse experiment) were processed by Seurat (version 3.1.5) (Butler et al., 2018). Genes expressed in at least one cell were reserved for further processing. The duplicates of three types of honeybee brain samples were merged by the Seurat merge function. In addition, cells with unique feature counts between 200 and 5000, the number of UMIs between 500 and upper limit (upper limit = Q3 + 1.5 ∗ (Q3 – Q1), Q1 and Q3 are the first and third quartiles) and less than 15% of mitochondrial genes were screened for further analysis.

After the filtered datasets were normalized using the default parameters, variable genes were found using the FindVariableFeatures function in Seurat with the parameters including selection.method = “vst”, nfeatures = 3000. Then, three types of honeybee brain datasets were integrated using the IntegrateData function in Seurat with dims = 1:30 parameter.

Then, the data were reduced and clustered using principal component analysis (PCA) and uniform manifold approximation and projection (UMAP) in Seurat. And Clustering of the data was processed using the FindClusters function with resolution = 1 parameter. Marks genes for each cluster were determined by Seurat FindAllMarkers function with parameters including only.pos = TRUE, min.pct = 0.1, logfc.threshold = 0.5, return.thresh = 0.01, test.use = “bimod”.

For reclustering of glia and Kenyon cells, cells set were selected by their respective marker genes, and analysis was carried out by the above method. The only difference was that the datasets of glia and Kenyon cells adopt 0.2 and 0.5 resolutions respectively. The information of all described genes in this study is summarized in Table S13.

#### Analysis of bulk RNA-seq

Bulk RNA-seq libraries were paired-end-sequenced. FastQC software (version 0.11.9) was used for quality control. Adapters were trimmed off with the Trimmomatic program (version 0.39) (Bolger et al., 2014). After filtering, the paired reads were aligned to the honeybee reference genome (assembly Amel_HAv3.1) using STAR software (version 2.7.9a) (Dobin et al., 2013). The STAR mapping produced reads were accurately quantified by the RSEM program (version 1.3.1) (Li and Dewey, 2011) to obtain count matrices about gene expression. The count matrices were processed by DEseq2 (version 1.32.0) (Love et al., 2014) R package.

#### Analysis of neuropeptides

The expression profiles of 27 reported bee neuropeptides were obtained from single cell RNA-seq and bulk RNA-seq. TBtools software (version 1.082) (Chen et al., 2020) was used to generate a heatmap.

#### Differentially expressed genes

For single cell RNA-seq data, differentially expressed genes were calculated by Seurat FindMarkers function with parameters including logfc.threshold = 0, min.pct = 0, test.use = “bimod”. The significance threshold was adjusted p value < 0.01, log2 foldchange of the average expression between the two groups >0.5 or < -0.5. For bulk RNA-seq data, adjusted p value < 0.01 and log2 foldchange >1 or < -1 in DEseq2 results were considered as differentially expressed genes.

#### Comparisons to bulk RNA-seq and single-cell RNA-seq

Pseudo-bulk normalized expressions of single-cell RNA-seq were obtained from three Seurat objects of forager, nurse, and queen. Compared with the normalized counts of bulk RNA-seq, the genes that were simultaneously detected were retained. The normalized expressions of single-cell RNA-seq and bulk RNA-seq were converted by log2 to calculate Spearman’s correlation coefficients using the stat_cor function from the ggpubr package.

#### Gene ontology enrichment analysis

The FindMarkers function of the Seurat R package was used to conduct data analysis. Genes with average log2 foldchange >0.5 and adjusted p value < 0.01 were considered as DE-Gs. GO term enrichment was performed using DAVID (Huang da et al., 2009a, b).

### Quantification and statistical analysis

Statistical analyses were performed using GraphPad Prism 8 software (version 8.2.1). Unpaired two side t-test was applied to determine whether the means of two populations were different, and differences were considered to be statistically significant at a value of p < 0.05.

## Data Availability

• Raw sequencing data have been deposited at GEO and are publicly available as of the date of publication. Accession numbers are listed in the key resources table. Raw micrograph data were deposited at online repository in Mendeley (https://doi.org/10.17632/xrd623n43s.1). • The codes used for analysis described in this study can be accessed at https://github.com/erroxml/Honeybee-scRNA-seq. • Any additional information required to reanalyze the data reported in this paper is available from the lead contact upon request.

## Supplemental information

Document S1. Figures S1–S5 and Tables S1–S3

Table S4. Predicted marker genes for all cell clusters, related to Figure 1

Table S5. Transcripts in Glial cells, related to Figure 1

Table S6. Transcripts in Kenyon cells, related to Figure 1

Table S7. Transcripts in Optic lobe cells, related to Figure 1.

Table S8. Transcripts in Olfactory projection neurons, related to Figure 1

Table S9. Transcripts in Hemocytes, related to Figure 1

Table S10. Transcripts in Undefined neurons, related to Figure 1

Table S11. DEGs from scRNA-seq, related to Figures 3 and 4

Table S12. DEGs from bulk RNA-seq, related to Figure 5

Table S13 Information of described genes, related to STAR Methods
