# Supplementary material for: Single-cell transcriptomic analysis of honeybee brains identifies vitellogenin as caste differentiation-related factor
Source: iScience. 2022 Jun 18;25(7):104643. doi: 10.1016/j.isci.2022.104643 (PMC9254125; doi:10.1016/j.isci.2022.104643)
Supplement: Document S1. Figures S1–S5 and Tables S1–S3 [file mmc1.pdf]

iScience, Volume *25*

Supplemental information

Single-cell transcriptomic analysis of honeybee brains identifies vitellogenin as caste differentiation-related factor

Wenxin Zhang, Liangliang Wang, Yinjiao Zhao, Yufei Wang, Chaoyang Chen, Yu Hu, Yuanxiang Zhu, Hao Sun, Ying Cheng, Qinmiao Sun, Jian Zhang, and Dahua Chen

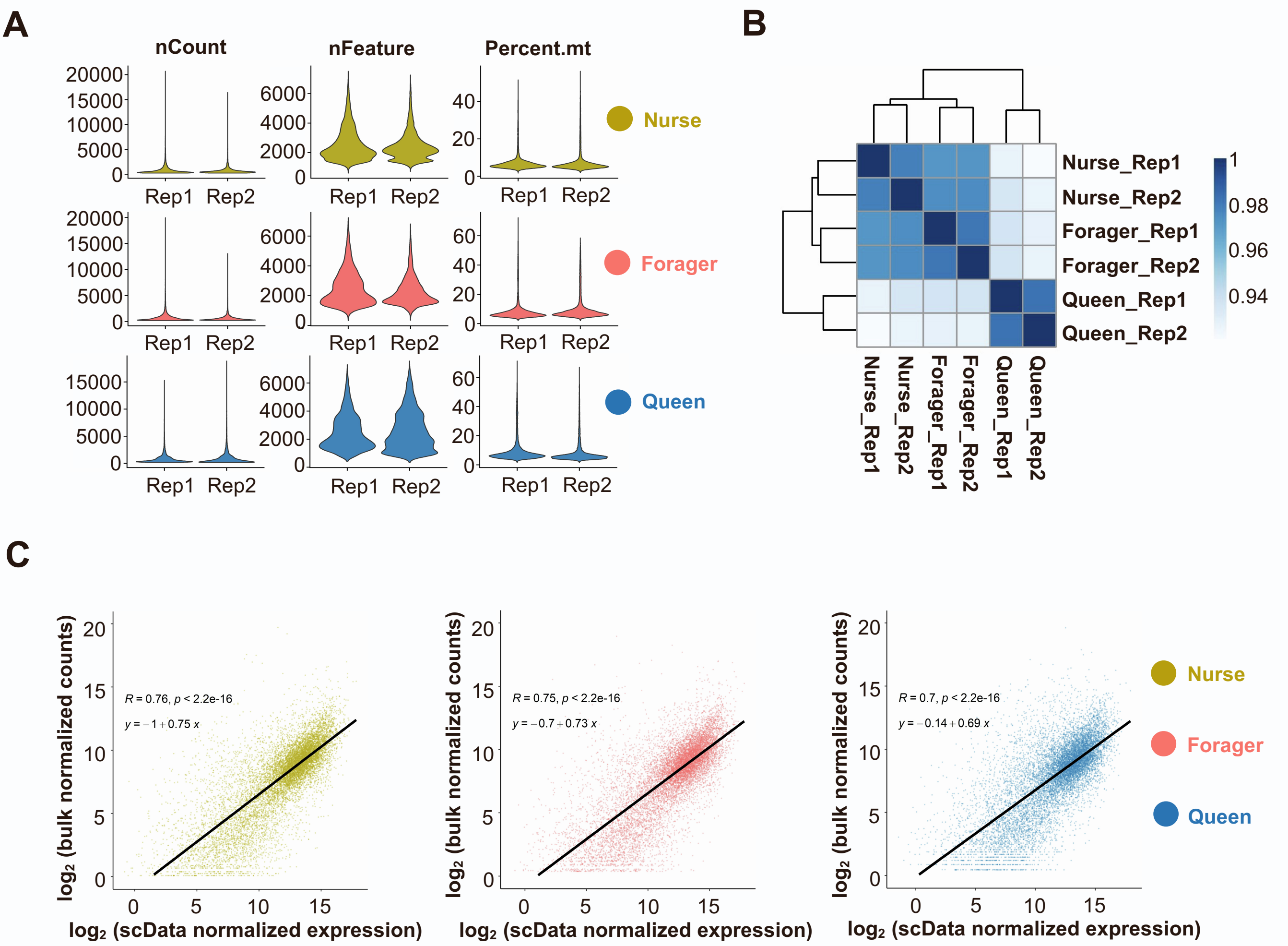
A
nCount
nFeature
Percent.mt
Nurse
Forager
Queen
Rep1
Rep2
B
Nurse_Rep1
Nurse_Rep2
Forager_Rep1
Forager_Rep2
Queen_Rep1
Queen_Rep2
1
0.98
0.96
0.94
C
R = 0.76, p < 2.2e-16
y = −1 + 0.75 x
R = 0.75, p < 2.2e-16
y = −0.7 + 0.73 x
R = 0.7, p < 2.2e-16
y = −0.14 + 0.69 x
log2 (bulk normalized counts)
log2 (scData normalized expression)

Figure S1. Cutoff parameters and correlation between single-cell RNA-seq and bulk RNA-seq.

Related to Figure 1.

(A) Violin plot showing the total number of molecules detected within a cell (nCount), the number of genes detected in each cell (nFeature), and the percent of mitochondrial transcripts (percent. mt) in each caste data. Dashed lines represent the following cutoffs: **nCount:** ⩾500 and up to Q3+1.5×(Q3-Q1), (Q1 and Q3 represent the first and third quartile, respectively), **nFeature:** ⩾200 and ⩽5,000, and **percent.mt:** ⩽15%;

(B) Clustered Spearman correlation matrix for different bulk RNA-seq replicates for three castes.

(C) Single-cell pseudo-bulk vs. bulk RNA-seq expression in the brain. Scatter plot showing the relationship between the expression level of each gene in this filtered single-cell RNA-seq datasets (log (normalized expression)) and the honeybee brain bulk RNA-seq datasets (log (normalized counts)). The regression line is in black and the Spearman's correlation coefficient (R), p-value (p), and equation of the regression line appear in the upper left corner.

Figure S2

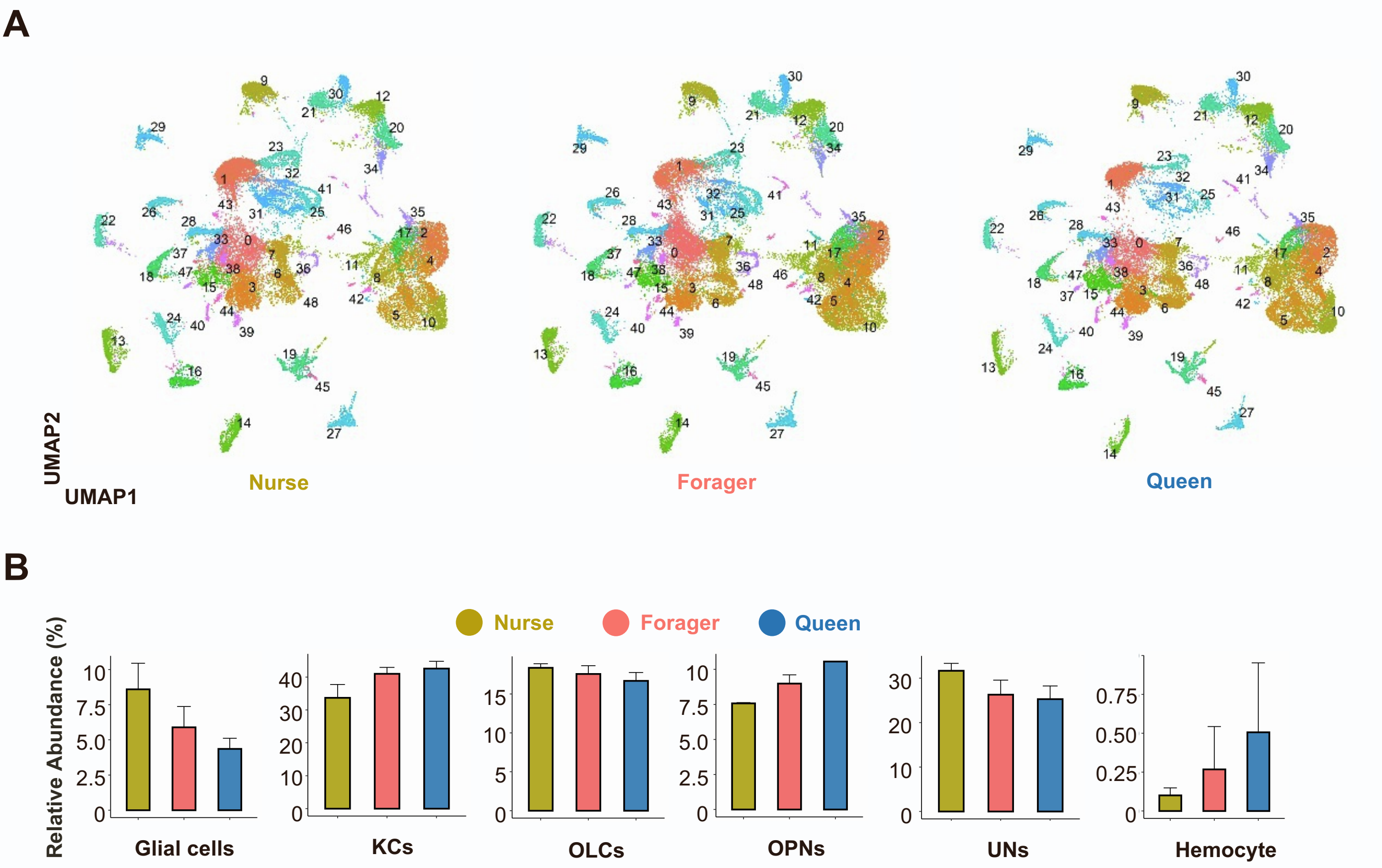
A
UMAP2
UMAP1
Nurse
Forager
Queen
B
Nurse
Forager
Queen
Relative Abundance (%)
Glial cells
KCs
OLCs
OPNs
UNs
Hemocyte

Figure S2. Single-cell transcriptome atlases of three castes.

Related to Figure 1.

(A) UMAP visualization of separated single cells in the brains of nurses, foragers, and queens, respectively.

(B) Percentages of each cell category among nurses, foragers, and queens. Y-axis: Average percent of subtypes across the three castes. Castes are shown in different colors. Each bar plot represents one subtype. Error bars represent + SD for two replicates.

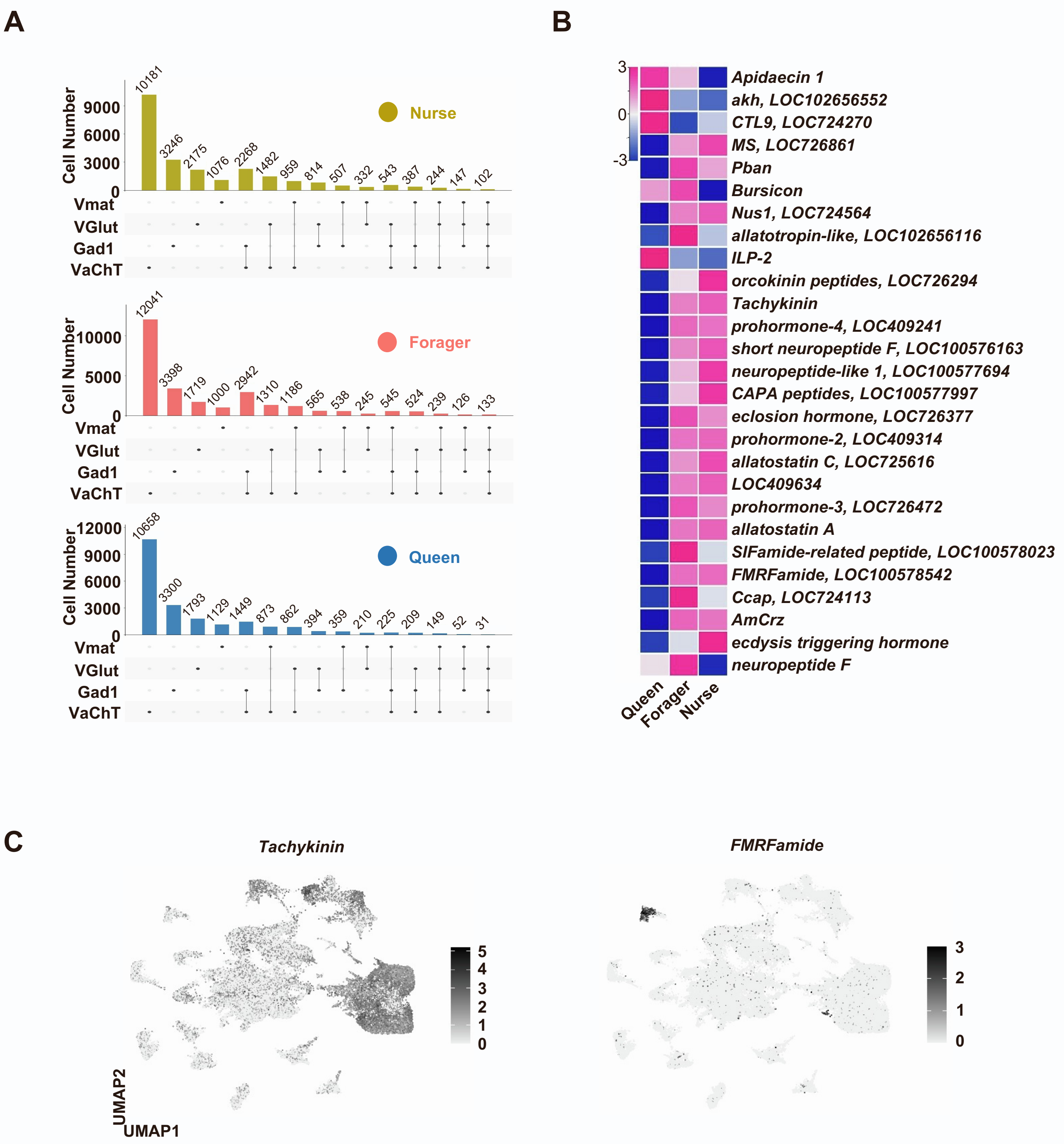
A
B
C
Nurse
Forager
Queen
Cell Number
Vmat
VGlut
Gad1
VaChT
10181
3246
2175
1076
2268
1482
959
814
507
332
543
387
244
147
102
12041
3398
1719
1000
2942
1310
1186
565
538
245
545
524
239
126
133
10658
3300
1793
1129
1449
873
862
394
359
210
225
209
149
52
31
Apidaecin 1
akh, LOC102656552
CTL9, LOC724270
MS, LOC726861
Pban
Bursicon
Nus1, LOC724564
allatotropin-like, LOC102656116
ILP-2
orcokinin peptides, LOC726294
Tachykinin
prohormone-4, LOC409241
short neuropeptide F, LOC100576163
neuropeptide-like 1, LOC100577694
CAPA peptides, LOC100577997
eclosion hormone, LOC726377
prohormone-2, LOC409314
allatostatin C, LOC725616
LOC409634
prohormone-3, LOC726472
allatostatin A
SIFamide-related peptide, LOC100578023
FMRFamide, LOC100578542
Ccap, LOC724113
AmCrz
ecdysis triggering hormone
neuropeptide F
Queen
Forager
Nurse
Tachykinin
FMRFamide
UMAP2
UMAP1

Figure S3. Expression of neurotransmitters and neuropeptides in three female groups.

Related to Figure 2.

(A) UpSet plots showing the co-expression of neurotransmitters in nurse, forager, and queen, respectively. Numbers represent the number of cells.

(B) Heatmaps showing expression patterns of 27 selected neuropeptides in bulk RNA-seq data from nurses, foragers, and queens, respectively. Expression is shown in magenta, intensity is proportional to the normalized expression levels.

(C) UMAP plots showing the distribution patterns of Tachykinin (left panel) and FMRFamide (right panel). Expression is shown in black, intensity is proportional to the normalized expression levels.

Figure S4

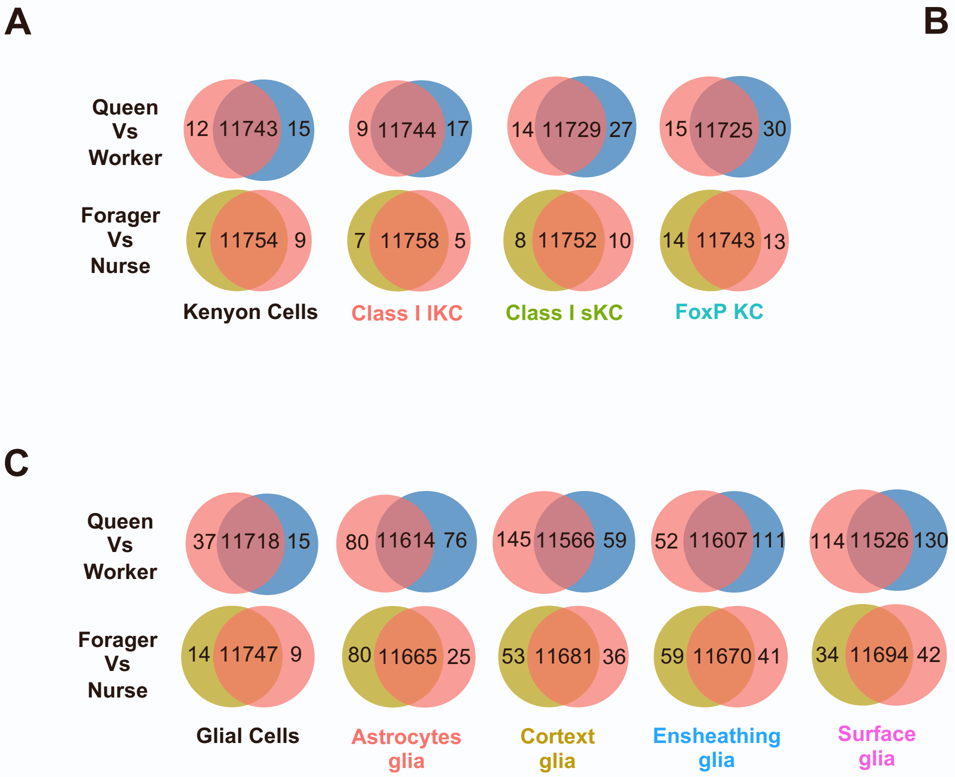
A
Queen Vs Worker
Forager Vs Nurse
12 11743 15
7 11754 9
Kenyon Cells
9 11744 17
7 11758 5
Class I lKC
14 11729 27
8 11752 10
Class I sKC
15 11725 30
14 11743 13
FoxP KC
B
Queen Vs Worker
Forager Vs Nurse
899 9964 482
59 11220 66
Bulk RNAseq
C
Queen Vs Worker
Forager Vs Nurse
37 11718 15
14 11747 9
Glial Cells
80 11614 76
80 11665 25
Astrocytes glia
145 11566 59
53 11681 36
Cortext glia
52 11607 111
59 11670 41
Ensheathing glia
114 11526 130
34 11694 42
Surface glia

Figure S4. Numbers of Differentially expressed genes in different castes and subcastes.

Related to Figures 3 and 4.

(A) Venn plots showing the number of overlapped and differentially expressed genes for Kenyon cell and its subtypes in different castes and subcastes. Top: comparison between different castes (queen vs. worker); Bottom: comparison between different subcastes (forager vs. nurse). Class I lKC: Class I large type Kenyon cells; Class I sKC: Class I small type Kenyon cells; FoxP KC: FoxP-expressed Kenyon cells

(B) Venn diagram showing the number of overlapped and differentially expressed genes in bulk RNA-seq data. Top: comparison between different castes (queen vs. worker); Bottom: comparison between different subcastes (forager vs. nurse).

(C) Venn plots showing the number of overlapped and differentially expressed genes for glia and its subtypes in different castes and subcastes. Top: comparison between different castes (queen vs. worker); Bottom: comparison between different subcastes (forager vs. nurse).

Figure S5

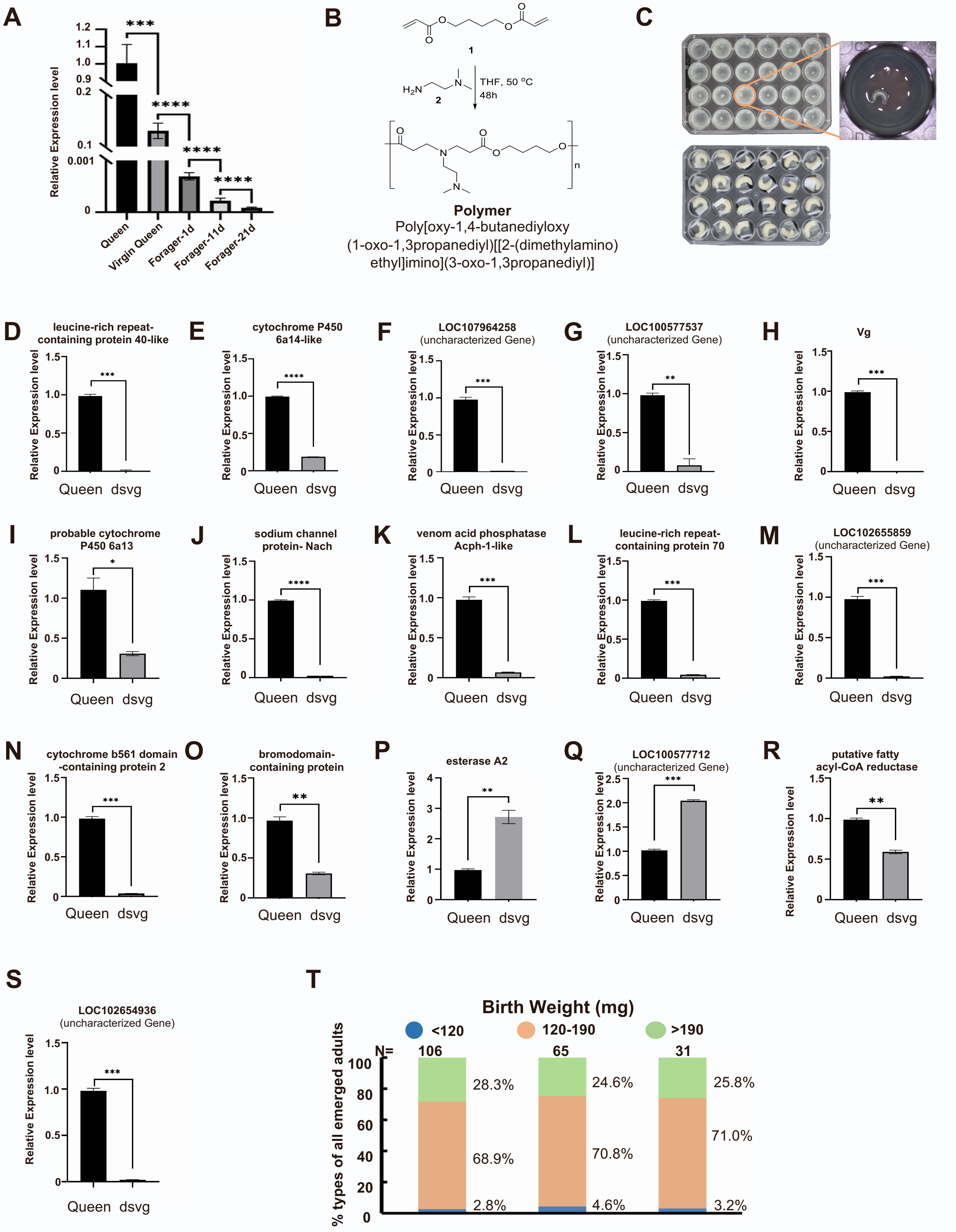

A
Relative Expression level
1.2
1.1
1.0
0.9
0.2
0.1
0.001
0
***
****
****
****
Queen
Virgin Queen
Forager-1d
Forager-11d
Forager-21d
B
1
2
THF, 50 °C
48h
n
Polymer
Poly[oxy-1,4-butanediyloxy
(1-oxo-1,3propanediyl)][[2-(dimethylamino)
ethyl]imino](3-oxo-1,3propanediyl)]
C
D
leucine-rich repeat-containing protein 40-like
E
cytochrome P450 6a14-like
F
LOC107964258 (uncharacterized Gene)
G
LOC100577537 (uncharacterized Gene)
H
Vg
I
probable cytochrome P450 6a13
J
sodium channel protein- Nach
K
venom acid phosphatase Acph-1-like
L
leucine-rich repeat-containing protein 70
M
LOC102655859 (uncharacterized Gene)
N
cytochrome b561 domain -containing protein 2
O
bromodomain-containing protein
P
esterase A2
Q
LOC100577712 (uncharacterized Gene)
R
putative fatty acyl-CoA reductase
S
LOC102654936 (uncharacterized Gene)
Relative Expression level
Queen
dsvg
T
Birth Weight (mg)
<120
120-190
>190
% types of all emerged adults
N=
106
65
31
100
80
60
40
20
0
28.3%
68.9%
2.8%
24.6%
70.8%
4.6%
25.8%
71.0%
3.2%

Figure S5. Knockdown of *vg* at the late larval stage does not affect caste differentiation

Related to Figure 5.

(A) Bar plot showing relative quantities of vg mRNA levels in honey bee brains from mature queens, virgin queens, 1-day foragers, 11-day foragers, and 21-day foragers. Bars are means±SD (three asterisks $P < 0.001$, four asterisks $P < 0.0001$).

(B) Synthesis method and the chemical structure of the nanomaterial polymer.

(C) The left upper panel shows the honeybee larvae at the early larval stage. The right panel shows the zoomed detail for a single larva on the left plate. The lower panel on the left shows the still larvae ready for pupation.

(D to S) Quantitative real-time PCR analysis for indicated genes in brains from newly emerged queens (higher birth weight (>190mg) adult from GFP-RNAi groups) and *vg* RNAi workers (lower birth weight (<120mg) adult from *vg*-RNAi groups). The bar plots show the relative expression levels of indicated genes. These genes are selected from the top highly expressed genes in queen brains according to bulk RNA-seq data. Most of the selected genes show consistent expression differences with bulk RNA-seq data.

(T) Statistics for three phenotypes of three experimental groups (blank, ds GFP, and ds vg-96hr). The phenotypes are colored according to different birth weight standards. The number of individuals and the percentage of each phenotype are shown.

Table S1. Primers for dsRNA template synthesis. Related to STAR METHODS.

**Oligonucleotides**	**SOURCE**	**IDENTIFIER**
dsRNA(*gfp*) Forward Primer: 5'-GAATTAATACGACTCACTATAGGGAGAaagctgaccctgaagttcatc-3' dsRNA(*gfp*) Reverse Primer: 5'-GAATTAATACGACTCACTATAGGGAGAtctcgttggggtctttgctc-3'	This paper	N/A
#1 dsRNA(*vg*) Forward Primer: 5'-GAATTAATACGACTCACTATAGGGAGAaacgtcaacatgttggacac-3' #1 dsRNA(*vg*) Reverse Primer: 5'-GAATTAATACGACTCACTATAGGGAGAtgaacggtccctcgttcttt-3'	This paper	N/A
#2 dsRNA(*vg*) Forward Primer: 5'-GAATTAATACGACTCACTATAGGGAGAatagccctttattcccaggg-3' #2 dsRNA(*vg*) Reverse Primer: 5'-GAATTAATACGACTCACTATAGGGAGAataccttcacctctttctgcc-3'	This paper	N/A
#3 dsRNA(*vg*) Forward Primer: 5'-GAATTAATACGACTCACTATAGGGAGAagaactgcctgttcagaaag-3' #3 dsRNA(*vg*) Reverse Primer: 5'-GAATTAATACGACTCACTATAGGGAGAaattcctcggtggtggaaac-3'	This paper	N/A
dsRNA(*IRP30*) Forward Primer: 5'-GAATTAATACGACTCACTATAGGGAGAggctcgctgataatatt-3' dsRNA(*IRP30*) Reverse Primer: 5'-GAATTAATACGACTCACTATAGGGAGActtcagcttcttcaacgct-3'	This paper	N/A
#1 dsRNA(*TOR*) Forward Primer: 5'-GAATTAATACGACTCACTATAGGGAGAatggggtttaaatcaatggga-3' #1 dsRNA(*TOR*) Reverse Primer: 5'-GAATTAATACGACTCACTATAGGGAGAcctgatcaggagttaatgaagg-3'	This paper	N/A
#2 dsRNA(*TOR*) Forward Primer: 5'-GAATTAATACGACTCACTATAGGGAGAagctagcagattatacttcgg-3' #2 dsRNA(*TOR*) Reverse Primer: 5'-GAATTAATACGACTCACTATAGGGAGAataactgcattacgcgttca-3'	This paper	N/A
#3 dsRNA(*TOR*) Forward Primer: 5'-GAATTAATACGACTCACTATAGGGAGAattttggagattgttttgaagtgg-3' #3 dsRNA(*TOR*) Reverse Primer: 5'-GAATTAATACGACTCACTATAGGGAGAgaattaaaagatcaacttgacgttg-3'	This paper	N/A

Table S2. Primers for qRT-PCR. Related to STAR METHODS.

**Oligonucleotides**	**SOURCE**	**IDENTIFIER**
RT-*RpL32* Forward Primer: 5’-ACTCGTCATATGTTGCCAACT-3’<br>RT- *RpL32* Reverse Primer: 5’-CCATGAGCAATTTCAGCACAA-3’<br>*RpL32* (as an internal reference of qRT-PCR in honeybee tissue samples)	This paper	N/A
RT-*vg* Forward Primer: 5’-AATTTGACGTATCCCTGGCTC-3’<br>RT-*vg* Reverse Primer: 5’-TCTGAACAGGCAGTTCTTGG-3’	This paper	N/A
RT-*IRP30* Forward Primer: 5’-ATCGAGGAGTGTCTGGCTCA-3’<br>RT-*IRP30* Reverse Primer: 5’-AGCCGATCTTTCTGTCCACG-3’	This paper	N/A
RT-*Tsf1* Forward Primer: 5’-CTCGAAGCGACCCGATCTTT-3’<br>RT-*Tsf1* Reverse Primer: 5’-CTCCTGCCAATCGTTCCACT-3’	This paper	N/A
RT-*GB17538* Forward Primer: 5’-TTGGTTCTCTTCTGTGCCGTT-3’<br>RT-*GB17538* Reverse Primer: 5’-CCTCAGGTTCCAATTCCGCT-3’	This paper	N/A
RT-*GB54690* Forward Primer: 5’-TGTATCCAGCAACAGCCTCG-3’<br>RT-*GB54690* Reverse Primer: 5’-TAGCCACGAAGCACAAGGAG-3’	This paper	N/A
RT-*GB55400* Forward Primer: 5’-GAACGTGGAGAACGTCCGAT-3’<br>RT-*GB55400* Reverse Primer: 5’-TGCACGCAGTATTTTTGGGC-3’	This paper	N/A
RT-*GB54511* Forward Primer: 5’-GAGGGACGAGGGAGGTAGTT-3’<br>RT-*GB54511* Reverse Primer: 5’-GACGGAAGGCGTGTGTCATA-3’	This paper	N/A
RT-*LOC100577073* Forward Primer:<br>5’-ACATTTCAACCCCCTACGAACAT-3’<br>RT-*LOC100577073* Reverse Primer:<br>5’-TTCTGTGAAAGTGCCCCGAG-3’	This paper	N/A
RT-*CSP3* Forward Primer: 5’-GCAGCTAACACGTCCAGCAA-3’<br>RT-*CSP3* Reverse Primer: 5’-CGAGCAGCCACAAGGACAAT-3’	This paper	N/A
RT-*GB52144* Forward Primer: 5’-ATGGGACAGCGATTACGTGG-3’<br>RT-*GB52144* Reverse Primer: 5’-AGGCATCAACCTCGTTGCTC-3’	This paper	N/A
RT-*GB41735* Forward Primer: 5’-ACATCGAGAACGTGGGCAAT-3’<br>RT-*GB41735* Reverse Primer: 5’-CCCGCGACTTTGGAATACCT-3’	This paper	N/A
RT-*Arr2* Forward Primer: 5’-TTGGTCGCAGAGGGTAAAGC-3’<br>RT-*Arr2* Reverse Primer: 5’-CACCTCCCAAAGTACCACAGTT-3’	This paper	N/A
RT-*RpL37A*- Forward Primer: 5’-TGACTGAGACGCGCAATGAA-3’<br>RT-*RpL37A*- Reverse Primer: 5’-CGTGCGTTTTGCCATTTTGC-3’	This paper	N/A
RT-*GB51409* Forward Primer: 5’-TCGAGTGTGCATCAGTGACC-3’<br>RT-*GB51409* Reverse Primer: 5’-GAGGACGTGTTTCGACGGAT-3’	This paper	N/A
RT-*GB40021* Forward Primer: 5’-TGTTGAAGGTCCCAAGACAGTAAG-3’<br>RT-*GB40021* Reverse Primer: 5’-TTTCCTCACCAACAGGATAGCG-3’	This paper	N/A
RT-*GB52597* Forward Primer: 5’-ATCATGAACAATGGAGGGGGC-3’<br>RT-*GB52597* Reverse Primer: 5’-TCGGACCGCAATTTCTTCGT-3’	This paper	N/A
RT-*prohormone2* Forward Primer: 5’-TACCCCAGGTGTGGGACAAT-3’<br>RT-*prohormone2* Reverse Primer: 5’-CCGCCGATTTCTCCGATCTT-3’	This paper	N/A
RT-LOC112935903 Forward Primer: 5’-CAATGTGCGAGAAGTTGCCG-3’<br>RT-LOC112935903 Reverse Primer: 5’-AACCACAGCTTCCAACGACA-3’	This paper	N/A

RT-LOC102654766 Forward Primer: 5'-GGTCTATGTCAACAATTCCAAGTGC-3' RT-LOC102654766 Reverse Primer: 5'-GTGGCTGAATTTCGTTGATGTAGC-3'	This paper	N/A
RT-*LOC107964258* Forward Primer: 5'-ATTTTGGGTGGGCAGTGCAG-3' RT-*LOC107964258* Reverse Primer: 5'-CTCGTGCAGCAATCGAAGATG-3'	This paper	N/A
RT-*LOC100577537* Forward Primer: 5'-TATCTCGTGGTAGGGACCTGG-3' RT-*LOC100577537* Reverse Primer: 5'-CGATATGCCGGTCGAAAGAGA-3'	This paper	N/A
RT-*LOC102653899* Forward Primer: 5'-CAATGTGCGAGAAGTTGCCG-3' RT-*LOC102653899* Reverse Primer: 5'-AACCACAGTTTCCGACGACAT-3'	This paper	N/A
RT-*LOC100578247* Forward Primer: 5'-AACAAATTTCAAACGCAACCAACAA-3' RT-*LOC100578247* Reverse Primer: 5'-TGCTCTGTTAATACATCCACTCCG-3'	This paper	N/A
RT-*LOC724418* Forward Primer: 5'-TCCCACCCTCTGAGAAGCAA-3' RT-*LOC724418* Reverse Primer: 5'-CAAAGAAGGAGTGCGTCGGA-3'	This paper	N/A
RT-*LOC107963996* Forward Primer: 5'-GCTGCGTGTATTGCATCTCG-3' RT-*LOC107963996* Reverse Primer: 5'-TCCAACCTTCTCCCTGTCCA-3'	This paper	N/A
RT-*GB40148* Forward Primer: 5'-GGTGCTCATGTGTCGAGTGA-3' RT-*GB40148* Reverse Primer: 5'-AAGTGATGCCCAGAATCCCG-3'	This paper	N/A
RT-*LOC102655859* Forward Primer: 5'-TCCGTGAATGGTGTAATATGGAACA-3' RT-*LOC102655859* Reverse Primer: 5'-GGAACACTGAAAATGGGCACAAA-3'	This paper	N/A
RT-*GB54794* Forward Primer: 5'-CGTGATACTTGAGCCACCGT-3' RT-*GB54794* Reverse Primer: 5'-ACGAGATCGACAAGCGTCTG-3'	This paper	N/A
RT-*GB43571* Forward Primer: 5'-TGATTCTGTGTTCCGAAGTGTGA-3' RT-*GB43571* Reverse Primer: 5'-TTCGATTGCTCTTTTTCAGGACAC-3'	This paper	N/A
RT-*GB48555* Forward Primer: 5'-ATCTGGAAACGTCACTGCGAT-3' RT-*GB48555* Reverse Primer: 5'-CCGCTCAGAATCCGTTCAGT-3'	This paper	N/A
RT-*LOC102654936* Forward Primer: 5'-TTATCGGACCAAAGGCGAGC-3' RT-*LOC102654936* Reverse Primer: 5'-TTCGAAAGCGCCATCAAGGT-3'	This paper	N/A
RT-*GB53516* Forward Primer: 5'-GTCTTAGGCGCGAGGCAATA-3' RT-*GB53516* Reverse Primer: 5'-GAGCTTTCGGCAGTGTGGAT-3'	This paper	N/A

Table S3. Probes for FISH. Related to STAR METHODS.

**Oligonucleotides**	**SOURCE**	**IDENTIFIER**
vg Antisense:<br>5'Quasar 570-CGUCCCGAUUCGCUGUCGCU-3'	This paper	N/A
idgf4 Antisense:<br>5'FAM -GCUGGGCCUUCAAGUCGCGG-3'	This paper	N/A
tsf1 Antisense:<br>5'Quasar 670-CGCGGCCACCAUCCGGACAG-3'	This paper	N/A
